# Differences in physical activity between weekdays and weekend days among U.S. children and adults: Cross-sectional analysis of NHANES 2011–2014 data

**DOI:** 10.1016/j.pmedr.2022.101892

**Published:** 2022-07-05

**Authors:** Quyen G. To, Robert Stanton, Stephanie Schoeppe, Thomas Doering, Corneel Vandelanotte

**Affiliations:** a Physical Activity Research Group, Appleton Institute, School of Health, Medical and Applied Sciences, Central Queensland University, Australia; b Cluster for Resilience and Wellbeing, Appleton Institute, School of Health, Medical and Applied Sciences, Central Queensland University, Australia

**Keywords:** Exercise, Pattern, MIMS, Correlate, Demographic, Anthropometric

## Abstract

This study investigates differences in physical activity between weekdays and weekend days, and its associations with sociodemographic and anthropometric characteristics in the U.S population using the NHANES 2011–2014 data. Physical activity was measured using ActiGraph GT3X+. Raw acceleration data were converted to Monitor-Independent Movement Summary units (MIMS-units) that account for inter-device variability and eliminates non-human movement. The daily MIMS-units were averaged for weekdays and weekend days. Demographic characteristics were self-reported. Height and weight were objectively measured. The sample included 6611 adults aged ≥ 20 years and 3520 children aged between 6 and 19 years. The results show that more MIMS-units were registered on weekdays compared to weekend days for adults (652, 95%CI = 533, 770) and children (889, 95%CI = 702, 1076). Factors associated with physical activity in adults (gender, age, marital status, ethnicity, education level, and weight status) were similar for weekdays and weekend days. In children, physical activity was associated with ethnicity, weight status, and age. The level of differences in physical activity between weekdays and weekend days among adults was larger for men, Hispanic Americans, and those with lower education levels. Differences among children were similar across subgroups, except for those aged 16–19 years who had smaller level of difference compared to those aged 12–15 years. In conclusion, adults and children were more active on weekdays than weekend days. The effects of structured days (i.e., weekdays) on physical activity appeared to be similar across most child subgroups but more heterogeneous for adult subgroups. Interventions increasing physical activity opportunities during weekend days are warranted.

## 1 Introduction

Physical activity is associated with multiple health benefits in adults and children. (Physical Activity Guidelines Advisory Committee, 2018) While numerous studies have examined a range of sociodemographic, anthropometric, and behavioural factors associated with physical activity, (Bauman et al., 2012, To et al., 2020, Sterdt et al., 2014) fewer studies have investigated associations of these factors with physical activity separately during weekdays and weekend days. Examining physical activity by weekdays and weekend days is necessary to understand how activities at work or school impact overall physical activity and can help decide whether specific intervention strategies are needed for weekend days compared to weekdays.

Recently, the Structured Days Hypothesis has been proposed. (Brazendale et al., 2017) It stated that the presence of structure, routine, and regulation within a day can positively influence children and adolescents’ physical activity, eating, and sleep behaviours. That is during structured days, children’s time is filled with “favorable activities” (e.g., scheduled physical education/ physical activity events, and regulated school meals) leaving less time for unhealthy activities (e.g., unlimited screen time). While the Structured Days Hypothesis is supported by many studies which have found that physical activity levels of children and adolescents are higher on weekdays compared to weekend days, (Brooke et al., 2014, Collings et al., 2014, To et al., 2018, Konharn et al., 2012, Duncan et al., 2010, Zosel et al., 2022, Brazendale et al., 2021) some studies found no significant difference. (Steele et al., 2010, Wilkin et al., 2006) Another study found more activity on weekend days, compared to weekdays in Chinese children. (Wong et al., 2015, Trost et al., 2000) found that children were more active but adolescents were less active on weekend days. (Trost et al., 2000) A potential limitation of many studies is the use of self-reported measures, (Zosel et al., 2022) which may limit generalizability to the general population. Moreover, differences in physical activity during weekdays and weekend days were mostly investigated for some characteristics such as age, gender, and weight status but less commonly for other characteristics such as poverty status and ethnicity. (Collings et al., 2014, To et al., 2018, Konharn et al., 2012, Duncan et al., 2010, Steele et al., 2010, Wong et al., 2015).

Among adults, fewer studies were conducted with mixed results. One study among 50–81 years old in England found participants more active on weekdays. (Schrempft et al., 2019) Using the 2003–2006 National Health and Nutrition Examination Survey (NHANES) data, it was found that only 3.2% of participants aged ≥ 18 years followed the ‘weekend warrior pattern’ (i.e., moderate-vigorous physical activity (MVPA) higher on weekend days). (Evenson et al., 2015) However, no difference in total step counts between weekdays and weekend days was found among office-based workers in England. (Smith et al., 2015) Another study found that physical activity was higher on weekdays for those walking to work, but similar for those travelling by car. (Audrey et al., 2014) These studies, however, did not examine associations of physical activity with other factors such as sociodemographic characteristics by weekdays and weekend days. (Schrempft et al., 2019, Evenson et al., 2015, Smith et al., 2015, Audrey et al., 2014).

This study, therefore, investigated the differences in physical activity between weekdays and weekend days, and its associations with sociodemographic and anthropometric characteristics using the NHANES 2011–2014 data. The findings can help develop strategies specific for weekdays and weekend days to improve the effectiveness of physical activity interventions.

## 2 Methods

### 2.1 Study design

This study used NHANES survey data collected annually by the US National Center for Health Statistics (NCHS). The survey uses a complex, four-stage, probability sampling method to select a nationally representative sample of approximately 5000 non-institutionalized participants across all ages from 15 counties each year. (Johnson et al., 2014) As physical activity was objectively measured using triaxial accelerometers in the NHANES 2011–2014, data from these years were used. Ethics approval was received from the NCHS Research Ethics Review Board (Protocol #2011–2017). Participants or their guardians provided informed consents for participation in the surveys.

### 2.2 Study population

**Fig. 1:**
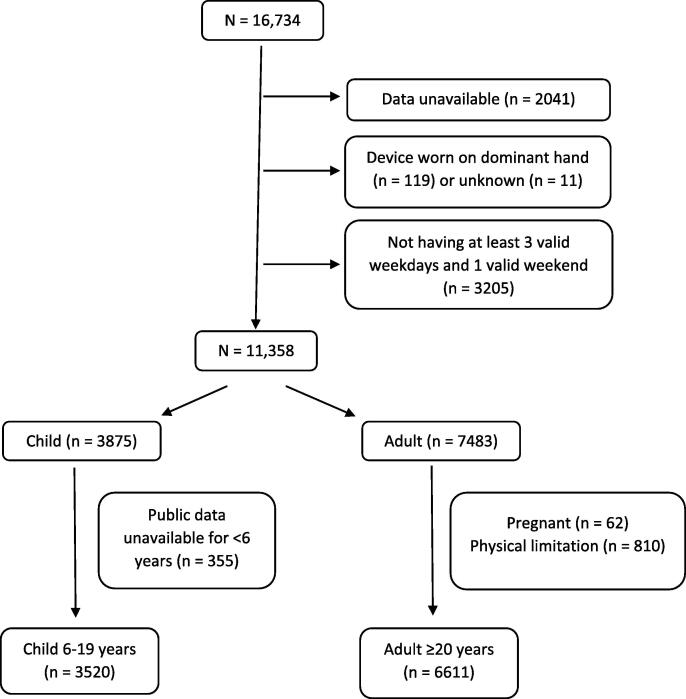
Flowchart of participant selection.

Among 16,734 participants, physical activity monitor data were available for 14,693 (Fig. 1). Participants were excluded if they wore the device on dominant wrist (n = 119) or wrist placement was unknown (n = 11). (Belcher et al., 2021) The device was worn for 9 days; however, the first and ninth days were incomplete and not used. A modified definition of a valid day from (Belcher et al., 2021) was used. (Belcher et al., 2021) Days with ≥ 1380 valid minutes (23 h), <72 min of non-wear time (<5% of 24 h), and < 17 h of sleep wear were considered valid. Participants without ≥ 3 valid weekdays and 1 valid WE were excluded (n = 3205) from analyses. Pregnant women (n = 62) and people who needed special equipment to walk (n = 810) were also excluded resulting in a total sample of 6611 adults aged ≥ 20 years and 3520 children aged between 6 and 19 years (data for children < 6 years were unavailable).

### 2.3 Measures

Demographic characteristics were self-reported. These included age, gender, marital status, ethnicity, family income for the adult and child samples, and education and marital status for the adult sample. Age groups used in the NHANES were “6–11 years”, “12–15 years”, or “16–19 years” for children; and “20–39 years”, “40–59 years”, or “≥60 years” for adults. Gender was male or female. Marital status was grouped into either “in a relationship” or “not in a relationship”. Ethnicity was categorized into “Mexican American/other Hispanic”, “Non-Hispanic White”, “Non-Hispanic Black/others” (including non-Hispanic multi-racial), and “Non-Hispanic Asian”. Education levels were “high school or below” or “above high school” for adults. Poverty income ratio was calculated by dividing family income by the poverty guidelines specific to the survey year, family size and geographic location. A ratio < 1 means that the family income is below the poverty line. Poverty income ratio were used as a categorical variable with cut-offs of 0 – 1.3 for low, >1.3 – 3.5 for middle, and > 3.5 – 5 for high. (Ogden et al., 2018).

Height and weight were measured by trained staff. (NHANES, 2011) Body Mass Index (BMI) was calculated by dividing weight(kg) by height(m) squared. The 2000 CDC growth charts were used to determine weight status of children aged < 20 years as not overweight/obese (BMI < 85th percentile) or overweight/obese (BMI ≥ 85th percentile). Adults were classified as not overweight/obese if BMI < 25 or overweight/obese if BMI ≥ 25.

### 2.4 Physical activity measurement

Physical activity was measured using an ActiGraph GT3X+ (ActiGraph, Pensacola, FL). The device is water resistant and was worn continuously, even while bathing and sleeping on participants’ nondominant wrist using a wristband for 9 days. (NHANES, 2022) The device was set to record the magnitude of acceleration at 80 Hz sampling frequency. Raw acceleration data were converted to Monitor-Independent Movement Summary units (MIMS-units) that account for inter-device variability and eliminate non-human movement. (John et al., 2019) Details on development and validation of the MIMS-unit are published elsewhere. (John et al., 2019) Briefly, the algorithm interpolates data to a consistent sampling rate, extrapolates to extend maxed-out signals, bandpass filters acceleration signals unrelated to voluntary human movement, and aggregates processed signals from each axis into a sum of MIMS-units that represents the total amount of movement activity. A higher MIMS value indicates a higher level of overall physical activity. Although there are no cut-off points for classifying these values into moderate or vigorous physical activity, reference values for some common activities are provided in the method paper. (John et al., 2019) For example, for wrist-worn devices, walking at 4.8 km/h and running at 8.8 km/h had values of about 25 and 140 MIMS-units/minute, respectively. Accordingly, a difference of 250 MIMS-units would mean a difference of 10 min walking at 4.8 km/h per day in this study.

Time periods of wake wear, sleep wear, and non-wear were estimated in three steps. In the first step, signal features were extracted from each of 1.5 min of data and a machine learning algorithm was used to classify each 30 s of the data into wake wear, sleep wear, or non-wear. Second, periods with minimum durations (i.e., <3min for wake wear, <10 min for sleep and non-wear) were filtered based on information from the prior and subsequent periods. If the prior and subsequent periods were not in the same predicted category, the predicted category of the current period was reassigned to that of the adjacent period with the higher confidence value. Third, orientation changes were used to reclassify longer periods from non-wear to sleep and vice versa to reflect periodic changes in body reorientation on the bed during sleep. (NHANES, 2020).

### 2.5 Data analysis

SAS software v9.4 was used for analyses. In compliance with the NHANES analytic guideline, (Chen et al., 2018) 4-year sampling weights were recalculated by dividing the 2-year weights by 2. Additionally, the sampling weights were post-stratified to match the U.S. civilian non-institutionalized population based on age, gender, and ethnicity. As percentages of missing values were about 8% for poverty income ratio variable and < 1% for the other variables, no imputation of missing data or further adjustment to the sampling weights were conducted. Survey procedures in SAS were used to account for the complex survey design. The Taylor series linearization variance estimation method was used for all analyses.

The daily MIMS-units were averaged separately for valid weekdays and weekend days. PROC SURVEYMEANS was used to calculate weighted MIMS-units and 95% confidence interval (CI) for each subgroup on weekdays, weekend days, and difference between weekdays and weekend days. PROC SURVEYREG was used to compare weighted averages of MIMS-units between subgroups. Differences in MIMS-units between subgroups were presented separately for weekdays and weekend days. In addition, differences in MIMS-units between weekdays and weekend days were compared between subgroups. A bivariate model (Model 1) and a multivariable model (Model 2) controlling for age, gender, marital status, ethnicity, education level, poverty income threshold, and weight status in the adult sample or age group, gender, ethnicity, poverty income threshold, and weight status in the child sample were conducted. Due to multiple comparisons, Tukey-Kramer adjustment was applied to variables with more than two categories. All p-values were two-sided and considered statistically significant if < 0.05.

## 3 Results

For all sample characteristics, adults and children were more active on weekdays than weekend days (between 1.9% and 9.9% for adults, 3.3% and 7.6% for children) (Table 1, Table 2). Among adults, the largest difference between weekdays and weekend days was 1600 MIMS-units (95%CI = 1336, 1863) for Mexican Americans/Other Hispanics; and the smallest difference was 256 MIMS-unit (95%CI = 28, 485) for those with high poverty income threshold. Among children, the largest difference was 1210 MIMS-units (779, 1642) for those aged 12–15 years; and the smallest difference was 479 MIMS-units for those aged 16–19 years. Overall, the difference for adults was 652 (95%CI = 533, 770) and for children was 889 (95%CI = 702, 1076).

**Table 1:** Weighted MIMS-units (95% confidence interval), difference between weekdays and weekend days, and percentage of difference over weekdays for adults.

	n	Weekday	Weekend	Difference	%
Total	6611	13,991 (13849, 14134)	13,339 (13166, 13513)	652 (533, 770)	4.7%
Age	6611				
20–39 years	2154	14,882 (14658, 15106)	14,268 (13977, 14558)	614 (367, 862)	4.1%
40–59 years	2343	14,334 (14120, 14548)	13,567 (13399, 13736)	767 (598, 935)	5.4%
≥60 years	2114	12,035 (11816, 12254)	11,507 (11221, 11792)	528 (381, 675)	4.4%
Gender	6611				
Male	3259	13,671 (13446, 13897)	12,815 (12541, 13089)	857 (653, 1060)	6.3%
Female	3352	14,304 (14199, 14409)	13,851 (13674, 14028)	453 (308, 597)	3.2%
Marital status	6609				
Not in a relationship	2653	13,857 (13692, 14022)	13,055 (12858, 13253)	802 (615, 989)	5.8%
In a relationship	3956	14,070 (13878, 14262)	13,505 (13309, 13700)	565 (397, 733)	4.0%
Ethnicity	6611				
Non-Hispanic White	2687	13,494 (13342, 13646)	13,020 (12829, 13211)	473 (303, 644)	3.5%
Non-Hispanic Black/Others	1675	14,141 (13873, 14409)	13,492 (13227, 13757)	649 (444, 854)	4.6%
Non-Hispanic Asian	803	13,979 (13602, 14356)	13,711 (13399, 14023)	268 (28, 508)	1.9%
Mexican American/Other Hispanic	1446	16,092 (15852, 16331)	14,492 (14214, 14770)	1600 (1336, 1863)	9.9%
Education	6609				
High school or below	2845	14,793 (14531, 15055)	13,652 (13418, 13886)	1141 (960, 1322)	7.7%
Above high school	3764	13,541 (13390, 13692)	13,162 (12984, 13340)	379 (246, 511)	2.8%
Poverty Income Threshold	6129				
Low (0–1.3)	2005	14,585 (14324, 14847)	13,714 (13445, 13983)	872 (636, 1107)	6.0%
Middle (>1.3 – 3.5)	2160	14,114 (13864, 14364)	13,178 (12895, 13461)	936 (755, 1117)	6.6%
High (>3.5 – 5.0)	1964	13,477 (13308, 13646)	13,221 (12984, 13458)	256 (26, 485)	1.9%
Weight status	6589				
Not overweight or obese	1997	14,404 (14156, 14653)	14,009 (13698, 14320)	395 (161, 629)	2.7%
Overweight or obese	4592	13,815 (13637, 13992)	13,055 (12874, 13235)	760 (666, 854)	5.5%

**Table 2:** Weighted MIMS-units (95% confidence interval), difference between weekdays and weekend days, and percentage of difference over weekdays for children.

	n	Weekday	Weekend	Difference	%
Total	3520	17,366 (17026, 17706)	16,477 (16103, 16851)	889 (702, 1076)	5.1%
Age	3520				
6–11 years	1884	20,107 (19794, 20420)	19,181 (18872, 19490)	926 (683, 1169)	4.6%
12–15 years	858	16,006 (15614, 16398)	14,796 (14239, 15352)	1210 (779, 1642)	7.6%
16–19 years	778	14,659 (14205, 15113)	14,180 (13605, 14755)	479 (130, 828)	3.3%
Gender	3520				
Boys	1773	17,466 (17055, 17878)	16,408 (15928, 16888)	1058 (787, 1330)	6.1%
Girls	1747	17,260 (16848, 17673)	16,549 (16126, 16972)	711 (504, 919)	4.1%
Ethnicity	3520				
Non-Hispanic White	841	17,361 (16826, 17896)	16,564 (15993, 17135)	797 (484, 1110)	4.6%
Non-Hispanic Black/Others	1157	17,326 (16976, 17675)	16,340 (15963, 16716)	986 (713, 1259)	5.7%
Non-Hispanic Asian	373	16,117 (15626, 16609)	14,943 (14395, 15492)	1174 (697, 1651)	7.3%
Mexican American/Other Hispanic	1149	17,670 (17231, 18109)	16,705 (16275, 17135)	965 (725, 1205)	5.5%
Poverty Income Threshold	3291				
Low (0–1.3)	1583	17,642 (17250, 18033)	16,871 (16462, 17280)	930 (693, 1166)	4.7%
Middle (>1.3 – 3.5)	1067	17,141 (16608, 17673)	16,162 (15602, 16722)	1123 (693, 1553)	7.1%
High (>3.5 – 5.0)	641	17,349 (16904, 17795)	16,525 (15925, 17125)	523 (202, 845)	3.6%
Weight status	3486				
Not overweight or obese	2215	17,558 (17144, 17973)	16,666 (16230, 17103)	770 (507, 1033)	4.4%
Overweight or obese	1271	17,100 (16738, 17463)	16,172 (15745, 16599)	979 (583, 1374)	5.7%

Younger adults were significantly more active with a higher MIMS value on both weekdays and weekend days compared to older adults in the adjusted model (Model 2, Table 3). Women, adults in a relationship, Hispanics, and non-overweight adults were significantly more active on weekdays and weekend days. Those with an education level above high school were significantly less active on weekdays and weekend days compared to those with a high school degree or below. Although those at middle poverty income threshold were significantly more active compared to high poverty income threshold on weekdays, no significant difference was found between low vs. high poverty income threshold groups, or on weekend days.

**Table 3:** Difference in MIMS-units on weekdays and weekend days, and weekdays vs. weekend days for adults.

	Weekday		Weekend		Weekday vs. Weekend	
	Model 1	Model 2^a^^,^^b^	Model 1	Model 2^a^^,^^b^	Model 1	Model 2^a^^,^^b^
Age						
20–39 years vs. ≥ 60 years	2847^***^ (2549, 3146)	2651^***^ (2322, 2980)	2761^***^ (2342, 3180)	2699^***^ (2298, 3099)	86 (−252, 424)	−48 (−425, 329)
40–59 years vs. ≥ 60 years	2299^***^ (1932, 2667)	2232^***^ (1867, 2597)	2060^***^ (1638, 2483)	2058^***^ (1625, 2492)	239 (−25, 502)	174 (−68, 415)
Gender						
Female vs Male	632^***^ (428, 836)	730^***^ (521, 940)	1036^***^ (739, 1333)	1103^***^ (825, 1382)	−404^**^ (−662, −146)	−373^**^ (−633, −113)

Marital status						
In a relationship vs. Not	213 (−21, 447)	485^***^ (235, 735)	449^***^ (266, 633)	710^***^ (532, 889)	−236 (−504, 31)	−226 (−472, 21)

Ethnicity						
Hispanic vs. NH Asian	2113^***^ (1502, 2725)	1664^***^ (973, 2355)	781^***^ (239, 1270)	736* (137, 1335)	1332^***^ (870, 1794)	928^***^ (470, 1385)
Hispanic vs. NH Black/others	1951^***^ (1521, 2381)	1433^***^ (1042, 1825)	1000^***^ (541, 1459)	645^**^ (153, 1138)	951^***^ (565, 1337)	788^***^ (415, 1162)
Hispanic vs. NH White	2598^***^ (2194, 3002)	1690^***^ (1272, 2109)	1472^***^ (1059, 1885)	842^***^ (325, 1358)	1126^***^ (685, 1567)	849^***^ (381, 1317)

Education						
Above high school vs. High school/below	−1252^***^ (−1561, −943)	−1060^***^ (−1374, −746)	−489^***^ (−712, −267)	−579^***^ (−884, −274)	−763^***^ (−955, −570)	−482^***^ (−688, −275)

Poverty Income Threshold						
Low vs. High	1109^***^ (702, 1515)	152 (−195, 499)	493* (15, 970)	−81 (−630, 469)	616^**^ (180, 1052)	233 (−256, 722)
Middle vs. High	637^***^ (328, 946)	304* (13, 594)	−44 (−420, 333)	−164 (−593, 265)	680^***^ (342, 1019)	468^**^ (108, 828)

Weight status						
Not overweight/obese vs. overweight/obese	589^***^ (284, 895)	555^***^ (276, 834)	955^***^ (628, 1281)	823^***^ (516, 1130)	−365^**^ (−572, −158)	−268* (−485, −50)

a complete cases used (n = 6107);

b model 2 includes age group, gender, marital status, ethnicity, education level, poverty income threshold, and weight status.

* p < 0.05, ^**^p < 0.01, ^***^p < 0.001.

Differences in MIMS-units between weekdays and weekend days were significantly smaller for women compared to men, those with an education above high school level compared to those with a lower educational level, and those not overweight compared to overweight/obese adults; but significantly larger for Hispanic compared to Non-Hispanic Asians, Non-Hispanic Black/others, and Non-Hispanic White, and those with middle compared to high poverty income threshold.

Compared to children aged 16–19 years, those aged 6–11 years and 12–15 years were significantly more active on weekdays; however, the difference was only significant on weekend days for those aged 6–11 years (Table 4). Non-Hispanic Asian children were significantly less active than Non-Hispanic White, Non-Hispanic Black/others, and Hispanic on weekdays and weekend days. Non-overweight children were significantly more active on weekend days. There was no significant difference in MIMS-units between boys and girls, or among children with different poverty income threshold. Differences in MIMS-units between weekdays and weekend days were significantly larger for those aged 12–15 years compared to 16–19 years but not significant for other comparisons.

**Table 4:** Difference in MIMS-units on weekdays and weekend days for children.

	Weekday		Weekend		Weekday vs. Weekend	
	Model 1	Model 2^a^^,^^b^	Model 1	Model 2^a^^,^^b^	Model 1	Model 2^a^^,^^b^
Age						
6–11 years vs. 16–19 years	5447^***^ (4880, 6014)	5530^***^ (4970, 6091)	5001^***^ (4259, 5742)	5145^***^ (4343, 5948)	447 (−61, 954)	385 (−144, 914)
12–15 years vs. 16–19 years	1347^***^ (728, 1965)	1393^***^ (786, 2000)	615 (−237, 1468)	736 (−144, 1615)	731* (120, 1343)	657* (22, 1293)
Gender						
Girls vs. boys	−206 (−672, 260)	−162 (−525, 200)	141 (−371, 653)	155 (−225, 536)	−347* (−660, −34)	−317 (−675, 40)

Ethnicity						
NH White vs. NH Asian	1244^**^ (296, 2191)	1309^***^ (508, 2111)	1620^***^ (650, 2591)	1769^***^ (759, 2780)	−377 (−1179, 425)	−460 (−1324, 404)
NH Black/others vs. NH Asian	1208^***^ (591, 1826)	1149^***^ (625, 1673)	1396^***^ (767, 2025)	1343^***^ (498, 2187)	−188 (−808, 433)	−194 (−939, 552)
Hispanic vs. NH Asian	1552^***^ (838, 2267)	1335^***^ (683, 1988)	1762^***^ (980, 2543)	1637^***^ (730, 2543)	−21 (−532, 489)	−302 (−1004, 401)

Poverty Income Threshold						
Low vs. High	292 (−301, 885)	282 (−202, 766)	346 (−378, 1070)	349 (−168, 866)	−54 (−657, 549)	−66 (−704, 572)
Middle vs. High	−209 (−786, 369)	−94 (−657, 469)	−363 (−1058, 332)	−224 (−922, 474)	154 (−607, 916)	130 (−674, 934)

Weight status						
Not OW/OB vs. OW/OB	458* (33, 883)	362 (−25, 749)	494* (44, 945)	383* (39, 727)	−37 (−389, 316)	−21 (−399, 357)

a complete cases used (n = 3262);

b Model 2 includes age group, gender, ethnicity, poverty income threshold, and weight status.

* p < 0.05, ^**^p < 0.01, ^***^p < 0.001.

## 4 Discussion

The findings showed that children were more active on weekdays than weekend days, which is consistent with previous studies. (Brooke et al., 2014, Collings et al., 2014, To et al., 2018, Konharn et al., 2012, Duncan et al., 2010, Zosel et al., 2022) The percentage of difference among children (5.1%) was also similar to that of 52 meta-analysed studies (5.1%). (Brooke et al., 2014) Adults were also found to be more active on weekdays than weekend days which is consistent with the findings from two previous studies. (Schrempft et al., 2019, Evenson et al., 2015) Other studies found no significant difference (Smith et al., 2015) or only significant for those walking to work (18.7% higher on weekdays). (Audrey et al., 2014) While there is lack of consensus for the minimal physical activity amount being clinically significant, (Loprinzi et al., 2016, Murakami et al., 2015) it is broadly agreed that any increase in physical activity leads to health benefits. (Physical Activity Guidelines Advisory Committee, 2018) These results provide evidence supporting the Structured Days Hypothesis that children are more active on structured days when more physical activity opportunities, e.g., attending physical education classes or travelling to school, are available. (To et al., 2020, Aibar et al., 2014) Furthermore, the results may suggest that Structured Days Hypothesis could be extended to adults who may be more active during weekdays due to travelling to work and work-related activities. (Audrey et al., 2014, Van Domelen et al., 2011).

Generally, factors associated with adults’ physical activity (including age, gender, marital status, ethnicity, education level, and weight status) were consistent across weekdays and weekend days. Age group and weight status were inversely associated with physical activity, which is consistent with the previous findings. (Bauman et al., 2012) Moreover, the finding that Hispanic adults were more active than other ethnic groups is consistent with NHANES 2003–2004 data. (Gay and Buchner, 2014) This may be due to occupational physical activity as studies have found Hispanic American are likely to have more physically demanding jobs. (Gay and Buchner, 2014) However, the associations of physical activity with gender, education, and marital status were inconsistent with the review of reviews. (Bauman et al., 2012) In the present analysis, women were more active than men. This may be explained by the use of wrist-worn accelerometers that better captured household activities in which women usually engaged more than men. (Bianchi et al., 2012) As women’s physical activity preferences differ from men (i.e., women prefer less competitive and vigorous activities), (van Uffelen et al., 2017) it is also possible that these activities may be better recorded by wrist-worn accelerometers. Although recent research using wrist-worn accelerometers have shown similar findings, (Belcher et al., 2021, Doherty et al., 2017, Wennman et al., 2019) more research is needed to identify the reasons. Additionally, participants with at least high school education were more active compared to those with higher education levels. Similarly, this could be due to occupational physical activity as those with lower education levels were more likely to have blue-collar jobs that require a higher physical activity level. (Kirk and Rhodes, 2011, Smith et al., 2016, Vandelanotte et al., 2015) While a study in Poland found that single urban adults were more likely to meet the physical activity guidelines than married adults, (Puciato and Rozpara, 2021) this study and others have found those in a relationship were more active. (Sobal and Hanson, 2010, Pettee et al., 2006) Although the personal commitments associated with being in a relationship may reduce the time couples engaged in physical activity, being in a relationship may also mean an increase in social support that could result in a more healthier and active lifestyle. (Bauman et al., 2012, Schoeppe et al., 2018) Overall, the inconsistency could be due to differences in cultural context, sample characteristics, and the use of different measurement tools (especially self-report and older models of waist-worn accelerometers) among studies.

Some studies found no association between age and physical activity among children, (Sallis et al., 2000, Van der Horst et al., 2007) whereas two of three reviews found inverse associations among adolescents. (Sallis et al., 2000, Van der Horst et al., 2007, Biddle et al., 2005) In this study, age group was inversely associated with physical activity levels during weekdays. However, the difference between 12–15 years and 16–19 years was not significant during weekend days. This finding suggests that activity patterns during weekend days were more similar among teenagers compared to younger children. Moreover, physical activity was not associated with children’s gender and poverty status which is inconsistent with the findings from previous reviews. (Bauman et al., 2012, Sterdt et al., 2014) The reasons for this finding are unclear. However, one explanation could be due to the use of waist vs wrist-worn accelerometers. While many studies with waist-worn accelerometers showed that boys were more active than girls, (Cooper et al., 2015, Troiano et al., 2008) the findings from wrist-worn accelerometry data are mixed. One study in the U.S. using wrist-worn ActiGraph GT3X + data from the 2014 National Cancer Institute’s Family Life, Activity, Sun, Health, and Eating Study showed that MVPA, calculated by four out of five data processing techniques, was not significantly different between boys and girls. (Kim et al., 2017) Another ActiGraph GT3X study in Scotland found that while boys spent more time on vigorous-intensity physical activity, they spent much less time on light-intense physical activity, and no difference in time spent on moderate-intense physical activity. (McLellan et al., 2020) However, wrist-worn Actigraph GT3X+ and GT9X data from a study in Hunter region of New South Wales, Australia still found that boys were more active than girls. (McCarthy et al., 2021) Given the complexity of dealing with accelerometry data (e.g., various data processing methods and device brands) and differences in sample characteristics across studies, more studies are needed to determine which placement position is better. However, it is worth noting that wrist-worn devices were found to significantly increase compliance due to being more comfortable and less embarrassing to wear (McLellan et al., 2018, Fairclough et al., 2016) and could result in better estimates of physical activity. (Crouter et al., 2015).

In contrast to Asian adults whose activity level was comparable to that of non-Hispanic White and Black, in the child sample, Asians were the least active in both weekdays and weekend days, which is consistent with studies in California, USA (Allen et al., 2007) and London, England. (Brodersen et al., 2007) One explanation could be that in Asian cultures, children are expected to spend much time on academic learning, including attending tutoring classes after school and during weekend days (To et al., 2021, Dawson, 2010) and therefore, may have less time to be active. This explanation is supported by evidence that Asian’s participation rates in major sports in the USA are much lower than other ethnic groups. (Bushnell, 2022) Given a lack of studies among Asian children, more research is needed so that interventions could be designed and implemented to improve their physical activity.

The level of differences in physical activity levels between weekdays and weekend days was smaller for women compared to men which suggests that women may engage in additional physical activities (e.g., household chores) during weekend days. Moreover, the large differences between weekdays and weekend days among Hispanic Americans compared to other ethnic groups and among those with lower education levels provide some support for our above explanation about the contribution of occupational activity during weekdays to total amount of physical activity. (Kirk and Rhodes, 2011, Smith et al., 2016, Vandelanotte et al., 2015) However, further research is needed to confirm these observations and identify other factors that may affect these patterns.

In contrast, the level of differences in physical activity between weekdays and weekend days was not significantly different between subgroups of children with the exception that those aged 16–19 years had smaller difference between weekdays and weekend days than those aged 12–15 years. These results may suggest that physical activity patterns between weekdays and weekend days are similar across subgroups of children although the patterns among those aged 16–19 years may be more similar to adults as a result of their transition into adulthood. This may also suggest that except for the age group of 16–19 years, the effects of the structured days on physical activity are similar across multiple subgroups but more research on this topic is needed.

Although a large number of reviews have been conducted to identify physical activity correlates in children and adults, (Bauman et al., 2012, Sterdt et al., 2014) majority of the reviewed studies used self-reported physical activity questionnaires which are subject to recall bias. Additionally, the relationship between these factors and physical activity may change over time as society and lifestyle behaviours change, e.g., the widespread use of electronic devices in every population group, the increasingly popularity of work-life balance, increasing mechanization of blue-collar jobs, and smaller family size due to low birth rates. Therefore, further research using objective measures is still needed to identify these associated factors in the current context.

This study has a large sample representative of the U.S. population. Moreover, NHANES is one of the highest quality surveys ongoing in the U.S. for>20 years. Another strength is that physical activity was objectively measured by accelerometers. However, this study does not include variables that could help better understanding the findings, e.g., type of transport and occupation, and sport participation. Additionally, the MIMS-unit is a new measure with no cut-points available to classify activity intensity or meeting of physical activity guidelines. Consequently, interpreting the practical significance of these results at this moment may be difficult. However, MIMS-unit values provided in the method paper could be used to assist with interpretation of the results in the adult samples. (John et al., 2019) Reference values for children are not available.

In conclusion, adults and children were more active on weekdays than weekend days. Factors associated with physical activity in adults (gender, age, marital status, ethnicity, education level, and weight status) were consistent for weekdays and weekend days. In children, physical activity was associated with ethnicity (consistently for weekdays and weekend days), weight status (significantly on weekend days but marginally for weekdays), and age (difference between those 12–15 years and 16–19 years was not significant for weekend days). The level of differences in physical activity between weekdays and weekend days among adults was larger for men, Hispanic Americans, those with lower education levels; and among children was similar across the subgroups, except for those aged 16–19 years who had smaller level of difference compared to those aged 12–15 years. Further research on the effects of structured days on children and adults’ physical activity, and ethnic differences in physical activity for adults and children is needed. Interventions providing adults and children more opportunities to engage in physical activity during weekend days may be more efficient. In addition, targeting groups with lower physical activity levels such as overweight/obese adults and Asian children, may help reduce health disparities.

## 5 Declarations

*Ethics approval and consent to participate:* This study used public data from the NHANES which has been approved by the NCHS Research Ethics Review Board (Protocol #2011–2017). Participants or their guardians provided informed consents for participation in the surveys.

## 6 Consent for publication

Not applicable.

## 7 Availability of data and materials

The data are publicly available at https://wwwn.cdc.gov/nchs/nhanes/Default.aspx.

## Funding

Not applicable.

## CRediT authorship contribution statement

**Quyen G. To:** Conceptualization, Writing – original draft. **Robert Stanton:** Writing – review & editing. **Stephanie Schoeppe:** Writing – review & editing. **Thomas Doering:** Writing – review & editing. **Corneel Vandelanotte:** Writing – review & editing.

## Declaration of Competing Interest

The authors declare that they have no known competing financial interests or personal relationships that could have appeared to influence the work reported in this paper.
